# Modified method of patency judgement using patency capsule prior to capsule endoscopy in clinical practice

**DOI:** 10.1038/s41598-022-18569-y

**Published:** 2022-08-22

**Authors:** Takahiro Miyazu, Satoshi Osawa, Satoshi Tamura, Shinya Tani, Natsuki Ishida, Tomoharu Matsuura, Mihoko Yamade, Moriya Iwaizumi, Yasushi Hamaya, Takahisa Furuta, Ken Sugimoto

**Affiliations:** 1 First Department of Medicine, Hamamatsu University School of Medicine, Hamamatsu, Japan; 2 Department of Endoscopic and Photodynamic Medicine, Hamamatsu University School of Medicine, 1-20-1 Handayama, Higashi-ku, Hamamatsu, 431-3192 Japan; 3 Department of Laboratory Medicine, Hamamatsu University School of Medicine, Hamamatsu, Japan; 4 Center for Clinical Research, Hamamatsu University School of Medicine, Hamamatsu, Japan

**Keywords:** Gastroenterology, Crohn's disease, Video capsule endoscopy

## Abstract

In 2012, Japan approved the use of a tag-less patency capsule (PC), which evaluates gastrointestinal patency before small-bowel capsule endoscopy (SBCE). This study aimed to evaluate the validity of our modification on the passage criteria for this PC in clinical practice. We retrospectively enrolled 326 consecutive patients who underwent PC examination before SBCE. If X-ray could not reveal the PC in the body during the judgement time (30–33 h after ingestion), we defined it as ‘estimated patency’ and performed SBCE. We employed plain computed tomography (CT) for the second judgement, as needed. The overall patency rate was 95.1%. By X-ray, 41 (12.6%) patients were judged to have ‘estimated patency’, and SBCE could be safely performed. Plain CT judgement was necessary in 106 patients (32.5%). One PC case had a residual coating film associated with stenosis in a patient with Crohn’s disease (CD), and one (0.3%) SBCE case had capsule retention resulting from false CT judgement. Multivariate analysis revealed that established CD and inpatient were factors related to no-patency. In conclusion, PC is useful for examining gastrointestinal patency, keeping in mind CT misjudgement. If PC was not found in the body via X-ray, performing SBCE as ‘estimated patency’ seemed appropriate.

## Introduction

Since small-bowel capsule endoscopy (SBCE) was introduced by Iddan et al.^1^, it has been widely used because of minimal invasiveness and ability to visualise the entire small-bowel mucosa for diagnosing small-bowel pathologies, such as obscure gastrointestinal bleeding (OGIB), small-bowel tumour and inflammation, including Crohn’s disease (CD) and nonsteroidal anti-inflammatory drug (NSAID)-induced small intestinal injury^2–6^. However, SBCE occasionally results in capsule retention proximal to stenosis in the gastrointestinal tract^7–9^. Meta-analysis showed capsule retention incidence was 2.2% (95% CI 1.5 – 2.8%), 3.6% (95% CI 1.7 – 8.6%) and 8.2% (95% CI 6.0 – 11.0%) in OGIB, suspected inflammatory bowel disease (IBD) and established IBD cases, respectively^8^. Balloon-assisted enteroscopy (BAE) or surgery should be performed to retrieve the capsule when conservative treatment with medications does not result in capsule excretion^10,11^. The Agile™ patency capsule (PC), an ingestible and dissolvable capsule with an external scanner, was developed and used in western countries to assess the functional patency of the small bowel while avoiding capsule retention^12–16^.

In Japan, the PillCam™ PC was initially available in 2012 as a tag-less Agile™ PC for safer gastrointestinal evaluation^3,17,18^. Passage criteria, which include judging the passage of PillCam™ PC within 30–33 h after ingestion, are recommended in the enteroscopy practice guidelines^19^. Upon excretion, the medical professional should inspect and palpate the PC to confirm an intact capsule or intact body. If the PC remains in the body but is detected in the large bowel via X-ray, the gastrointestinal tract is considered patent. However, the entry of the PC into the large bowel should be further verified by computed tomography (CT)^20^, X-ray tomography^21^ or abdominal ultrasonography^22^, as necessary. Recently, its safety and usefulness have been reported in larger-scale prospective studies in Japan^23^. However, the methods may need to be modified in daily clinical practice. In fact, some modifications of the passage criteria suitable for daily clinical use were reported to increase the possibility to perform SBCE safely^24,25^.

However, the insights into how to deal with invisible PC excretion via X-ray during the judgement time (30–33 h after ingestion) and which imaging modality should be used for the second examination after confirming PC location by X-ray remain unknown. In this study, if PC was not detected in the body via X-ray during the judgement time, we considered it as ‘estimated patency’ before performing SBCE. Plain CT was used as the second imaging tool for judgement. We aimed to evaluate the validity of our modification on the passage criteria suitable for daily clinical practice and evaluated the safety and usefulness of PC in our consecutive cases.

## Results

### Patient characteristics

We enrolled 326 patients, with 198 males and 128 females (Table 1). The mean age was 52.2 ± 22.2 years. The most common reason for examination was OGIB (117, 35.9%), followed by established CD (75, 23.0%), other inflammatory diseases (29, 8.9%), abdominal pain (27, 8.3%), small-bowel tumour (20, 6.1%) and suspected CD (16, 4.9%). History of abdominal surgery, inpatient or outpatient, comorbidities, constipation and use of NSAIDs or LDA are shown in Table 1.

**Table 1:** Patients’ characteristics.

Numbers of patients	326
Sex, male/female	198/128
Age, mean ± SD (range), years	52.2 ± 22.2 (3–88)
Inpatient/outpatient	126/200
History of abdominal surgery, n (%)	108 (33.1)
**Reason for examination, n (%)**	
OGIB	117 (35.9)
Crohn’s disease, overall	91 (27.9)
Crohn’s disease, established	75 (23.0)
Crohn’s disease, suspected	16 (4.9)
Other inflammatory diseases	29 (8.9)
Abdominal pain	27 (8.3)
Small-bowel tumour	20 (6.1)
Intestinal obstruction	5 (1.5)
Others	37 (11.3)
Diabetes mellitus	51 (15.6)
Haemodialysis	19 (5.8)
Constipation	47 (11.3)
NSAIDs, LDA	64 (19.6)

*LDA* low-dose aspirin, *NSAIDs* nonsteroidal anti-inflammatory drugs, *OGIB* obscure gastrointestinal bleeding, *SD* standard deviation.

### Patency evaluation

**Figure 1:**
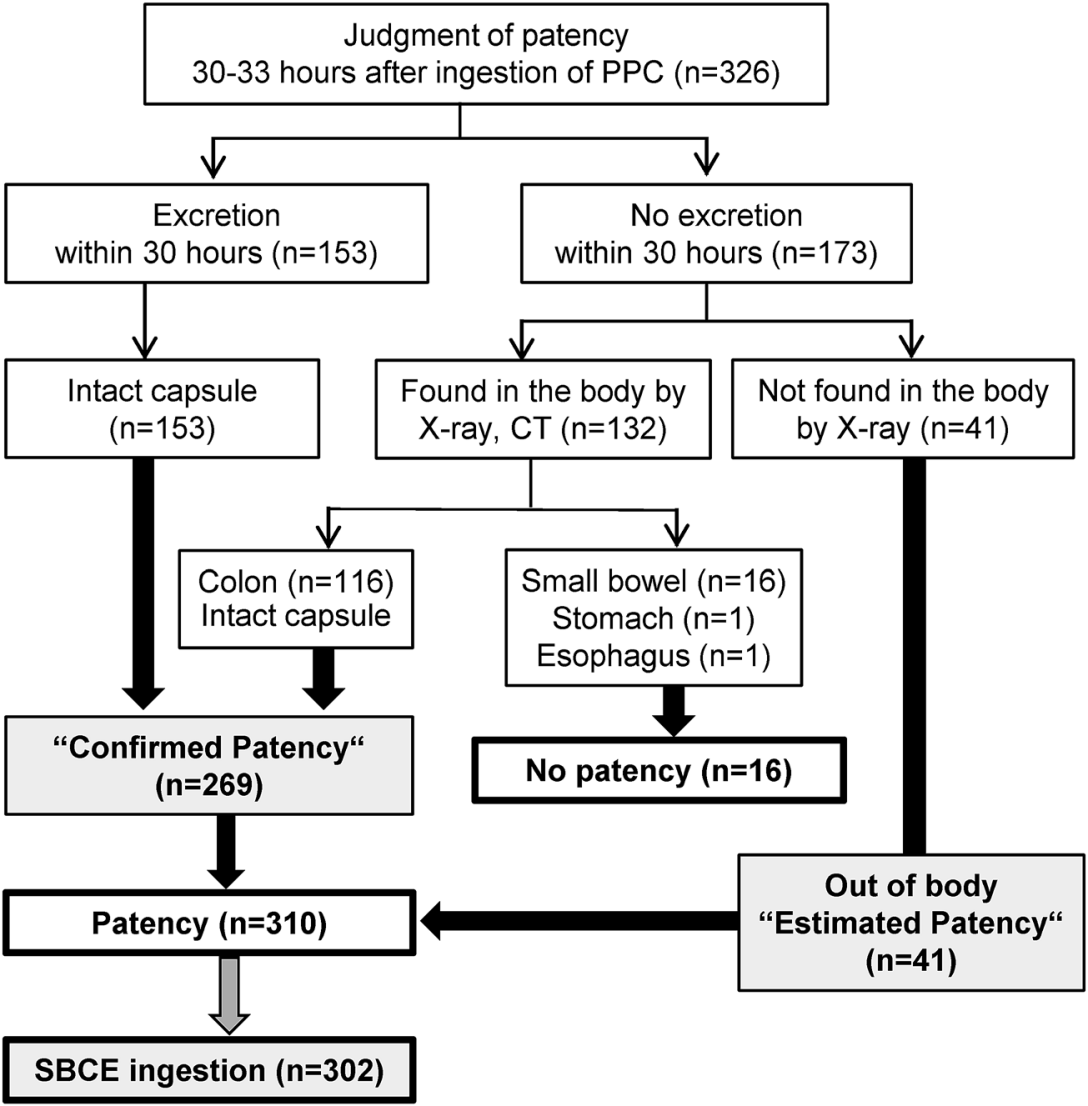
Study flow diagram.

Figure 1 presents the flow diagram of the study. Among 326 participants, 153 (46.9%) patients had confirmed patency by intact capsule recovery. In 41 (12.6%) patients, the PC could not be detected by X-ray during the judgement time, thereby judged as ‘estimated patency’. In 106 (32.5%) of 132 patients, plain CT judgement was needed when the PC was confirmed inside the body within 30–33 h after ingestion by X-ray. Finally, 16 patients (4.9%) had no patency and were excluded from SBCE. The overall patency rate was 95.1% (310/326) (Table 2).

**Table 2:** Results of the patency capsule procedure.

Overall patency, n (%)	310 (95.1)
Confirmed patency, n (%)	269 (82.5)
Estimated patency, n (%)	41 (12.6)
CT judgement, n (%)	106 (32.5)
No patency, n (%)	16 (4.9)
**Adverse events, n (%)**	**3 (0.9)**
Retention of the coating film	1 (0.3)
Abdominal pain	1 (0.3)
Nausea, vomiting	1 (0.3)
Intestinal obstruction	0
Perforation	0
Capsule aspiration	0
Allergic reaction	0

*CT* computed tomography.

### Factors associated with PC excretion within 30 h

Factors associated with PC excretion within 30 h are presented in Table 3. Univariate analysis showed that age, OGIB, inpatient, diabetes mellitus, haemodialysis and constipation significantly contributed to excretion inability within 30 h (*P* < 0.05). In multivariate analysis, eight factors were analysed, and among them, age, female and inpatient were the independently influencing factors associated with PC excretion within 30 h (*P* < 0.05) (Table 3).

**Table 3:** Univariate and multivariate logistic regression analyses of factors associated with excretion within 30 h.

Factor	Univariate analysis			Multivariate analysis		
	Crude OR	95% CI	*P* value	Adjusted OR	95% CI	*P* value
Age	0.978	0.968–0.989	0.0001	0.970	0.970–0.997	0.0139
Sex, F	0.648	0.412–1.020	0.0596	0.541	0.329–0.891	0.0157
OGIB	0.447	0.281–0.710	0.0006	0.680	0.377–1.230	0.2020
CD, established	1.600	0.930–2.770	0.0893	0.626	0.322–1.220	0.1670
CD, suspected	1.530	0.518–4.500	0.4430			
Inflammatory diseases	1.570	0.692–3.570	0.2800			
Abdominal pain	0.838	0.379–1.850	0.6620			
Small-bowel tumour	1.630	0.611–4.370	0.3280			
Intestinal obstruction	1.020	0.168–6.200	0.9820			
Inpatient	0.424	0.268–0.670	0.0002	0.381	0.226–0.643	0.0003
History of surgery	0.985	0.616–1.570	0.9480			
Diabetes mellitus	0.339	0.183–0.630	0.0006	0.610	0.298–1.250	0.1760
Haemodialysis	0.374	0.143–0.978	0.0448	1.000	0.341–2.940	0.9990
Constipation	0.363	0.192–0.686	0.0018	0.544	0.264–1.120	0.0997
NSAIDs	1.390	0.578–3.360	0.4600			

*CD* Crohn’s disease, *CI* confidence interval, *NSAID* nonsteroidal anti-inflammatory drug, *OGIB* obscure gastrointestinal bleeding, *OR* odds ratio.

### Factors associated with non-confirmation of patency

We found 16 patients whose PC did not confirm patency. Table 4 summarises the patient characteristics, and Supplementary Table S1 lists the details of individual clinical information. Eight patients had established CD. Univariate analysis showed that established CD was significantly associated with non-confirmation of patency (*P* < 0.05). In multivariate analysis, six factors were analysed, and among them, established CD and inpatient were the independently influencing factors associated with non-confirmation of patency (*P* < 0.05) (Table 5). The patency rates in established CD patients and inpatients were 89.3% (67/75) and 92.8% (117/126), respectively.

**Table 4:** Characteristics of the no patency.

Numbers of patients	16
Sex, male/female	11/5
Age, mean ± SD (range), years	51.4 ± 19.4 (21–81)
Inpatient/outpatient	8/8
History of abdominal surgery, n (%)	8 (50.0)
**Reason for examination, n (%)**	
OGIB	3 (18.8)
Crohn’s disease, established	8 (50.0)
Crohn’s disease, suspected	0 (0.0)
Other inflammatory diseases	2 (12.5)
Abdominal pain	0 (0.0)
Small-bowel tumour	1 (6.3)
Intestinal obstruction	0 (0.0)
Others	2 (12.5)
Diabetes mellitus	2 (12.5)
Haemodialysis	2 (12.5)
Constipation	2 (12.5)
NSAIDs, LDA	1 (6.3)

*LDA* low-dose aspirin, *NSAIDs* nonsteroidal anti-inflammatory drugs, *OGIB* obscure gastrointestinal bleeding, *SD* standard deviation.

**Table 5:** Univariate and multivariate logistic regression analyses of factors associated with intestinal patency.

Factor	Univariate analysis			Multivariate analysis		
	Crude OR	95% CI	*P* value	Adjusted OR	95% CI	*P* value
Age	1.000	0.980–1.020	0.8730	0.982	0.9520–1.010	0.2300
Sex, F	1.450	0.491–4.270	0.5030	1.180	0.3800–3.650	0.7760
OGIB	2.520	0.703–9.030	0.1560	2.250	0.4790–10.60	0.3040
CD, established	0.276	0.010–0.762	0.0013	0.215	0.0552–0.840	0.0270
Other IDs	0.668	0.144–3.090	0.6060			
Small-bowel tumour	0.979	0.123–7.810	0.9840			
Inpatient	0.472	0.171–1.300	0.1460	0.298	0.096–0.919	0.0035
History of surgery	0.476	0.174–1.310	0.1490	0.868	0.277–2.730	0.8090
Diabetes mellitus	1.310	0.290–5.960	0.7230			
Haemodialysis	0.406	0.009–1.930	0.2580			
Constipation	1.190	0.261–5.410	0.8230			

*CD* Crohn’s disease, *CI* confidence interval, *IDs* inflammatory diseases, *OGIB* obscure gastrointestinal bleeding, *OR* odds ratio.

### Adverse events

One of the adverse events caused by PC was the presence of residual coating film associated with stenosis in patients with CD. It was removed by double-balloon enteroscopy and treated by endoscopic balloon dilation. The mild adverse events were abdominal pain and vomiting, which were found in two patients separately (Supplementary Table S1).

### SBCE results

**Figure 2:**
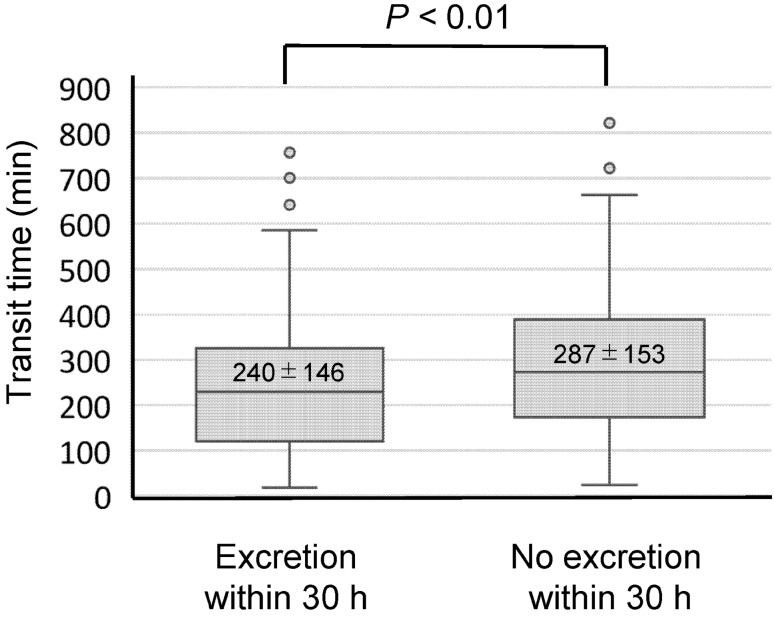
PC excretion within 30 h was associated with SBCE transit time. The transit time of the small-bowel capsule endoscopy (SBCE) was compared between the excretion and nonexcretion groups within 30 h.

**Figure 3:**
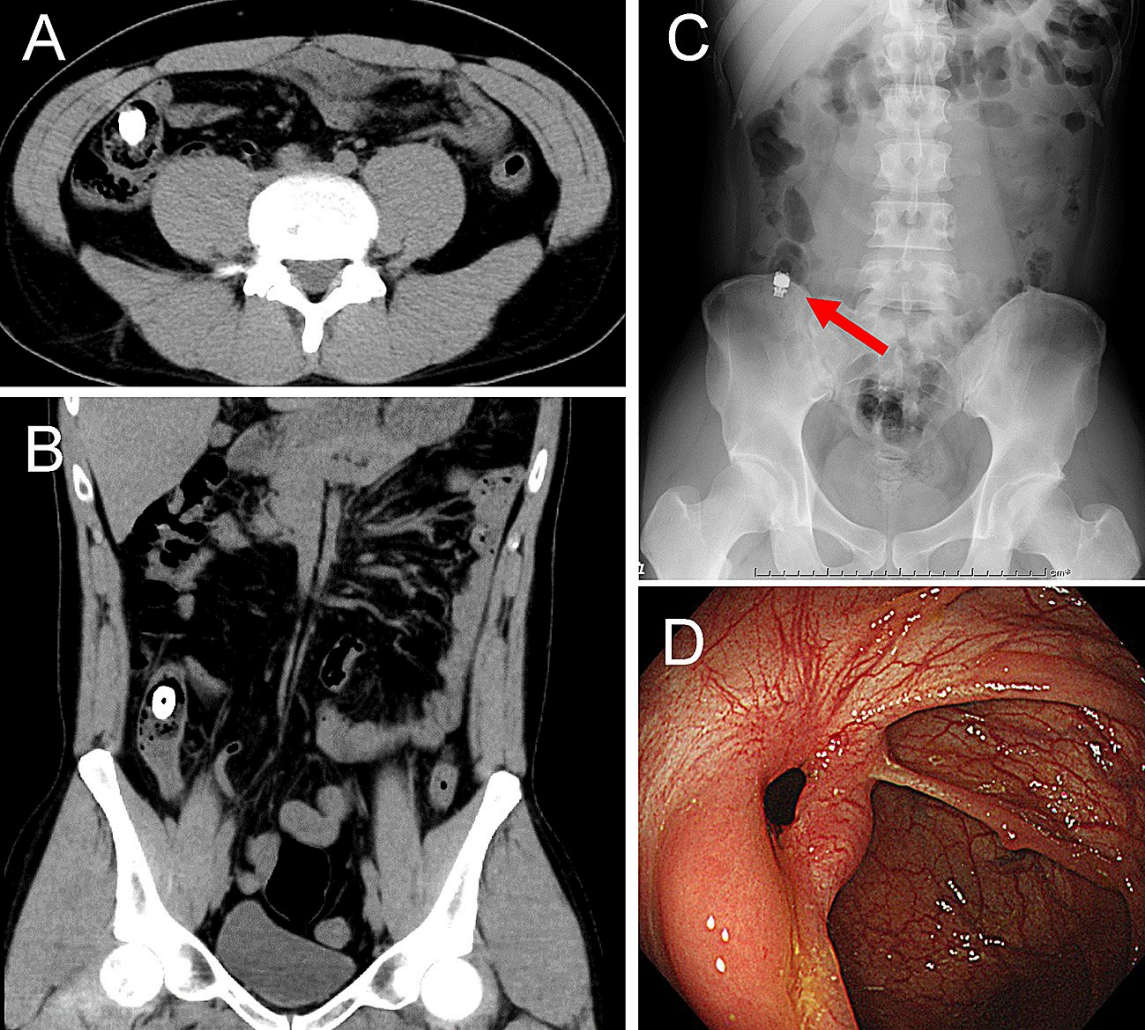
SBCE retention case caused by CT misjudgement of PC in a patient with Crohn’s disease. Plain computed tomography (CT) images show that the PC seemed to be in the ascending colon; axial CT image (**A**), coronal CT image (**B**). X-ray scan confirms SBCE retention (arrow) in the terminal ileum after 2 weeks (**C**). Colonoscopic image confirms ileocecal valve stenosis, which was treated with balloon dilatation (**D**).

SBCE was performed within 7 d in 302 patients (92.6%) who showed confirmed and estimated patency by PC examination. Table 6 summarises the SBCE results. The rate at which the entire small bowel could be observed was 93.1% (mean small-bowel transit time, 257 ± 150 min). The transit time of the SBCE was significantly shorter in the PC excretion group within 30 h (Fig. 2). The entire small bowel observation rate in patients with excretion within 30 h was 94.2% (180/191), whereas that in patients with no excretion in 30 h was 91.0% (101/111). There was no statistical difference between two groups (*P* = 0.348). Only one (0.3%) patient experienced capsule retention caused by a false CT judgement (Fig. 3).

**Table 6:** Results of the SBCE procedure.

SBCE examination, n (%)	302 (92.6)
Total small-bowel observation, n (%)	281 (93.1)
**Transit time, min, median (IQR)**	
Stomach	27.0 (9–80)*
Small bowel	238 (140–344)*
**Positive findings, n (%)**	223 (73.8)
Ulceration, erosions	140 (46.4)
Vascular lesions	51 (16.9)
Neoplasms	44 (14.6)
Others	13 (4.3)
**Adverse events, n (%)**	
Capsule retention	1 (0.3)
Capsule aspiration	0 (0.0)

*SBCE* small-bowel capsule endoscopy, *IQR* interquartile range.

## Discussion

In this study, we examined the validity of our modification on the passage criteria suitable for daily clinical practice and evaluated the safety and usefulness of PC in our consecutive patients. Our main findings were as follows. The overall patency rate was 95.1%, 41 (12.6%) patients were judged as having estimated patency, and SBCE was performed safely. In addition, 32.5% of the participants needed CT judgement. According to our passage criteria, one (0.3%) SBCE case resulted in capsule retention because of a false CT judgement. Multivariate analysis revealed that established CD and inpatient were the factors independently related to non-confirmation of patency (*P* < 0.05). Therefore, our modification on the passage criteria tailored for daily clinical practice seemed to be reasonable, keeping in mind CT misjudgement.

One of the main purposes of this study was to determine whether ‘estimated patency’ without retesting is valid in identifying patency for SBCE. Premature dissolution of the Agile™ patency device was reported to occur, with at least an incidence of 1.3% in Europe, possibly the cause of unexpected capsule retention^26^. This event was recognised by the detection of a persistent radiofrequency signal after radiological imaging had failed to identify the patency device. In Japan, patients who need to undergo a patency-testing procedure can avail insurance if an intact capsule or intact body with an eroded timer plug is directly detected or if PC entry into the colon is confirmed by X-ray or another imaging modality at 30–33 h after ingestion^19^. However, PC retesting is clinically difficult because it impairs the proper timing of the examination and patient acceptability. In this study, no case of SBCE retention in ‘estimated patency’ was found, suggesting the validity of our judgement method.

Another purpose of this study was to determine the validity of plain CT judgement after confirming the location of the PC inside the body. Candidates for the second imaging modality include plain or contrast-enhanced CT^27,28^, low-dose CT^20^, X-ray tomography^21^, abdominal ultrasonography^22^, air enema and magnetic resonance imaging. Among them, plain CT is an objective method and is the easiest to use in Japan, although it exposes patients to radiation. In this study, capsule retention occurred in one (0.3%) patient. This result seems to agree with the results of a recent large-scale multicentre study in Japan^23^. Our results suggested that the choice of plain CT as the second imaging modality is nearly valid. However, a misjudgement of CT was observed in one case. Other studies also revealed that CT misjudgement would be a non-negligible reason for SBCE retention after PC examination^23,25^. Physicians performing PC examinations should be aware of this issue.

Moreover, multivariate analysis indicated that age, female and inpatient were the factors independently associated with PC excretion within 30 h, and established CD and inpatient were the factors independently related to non-confirmation of patency (*P* < 0.05). CD is a recognised factor related to non-confirmation of patency, consistent with our results^29^. However, many reports indicated that inpatients are related to transit time but not to non-confirmation of patency. Although the exact reason for this discrepancy remains unknown, our results showed that inpatients were more likely to have organic disorders, such as radiation enteritis and NSAID-induced small-bowel mucosal injury. In addition, patients with impaired functional passage of the upper gastrointestinal tract might have been included. Physicians should also be aware that SBCE has a longer small-bowel transit time in the elderly and women, regardless of having non-confirmation of patency.

Although the safety and usefulness of PC have been reported in larger-scale prospective studies in Japan^23^ and European countries^30^, the methods may need to be modified in daily clinical practice. The European Society of Gastrointestinal Endoscopy (ESGE) has published evidence-based clinical and technical reviews^5^, whereas variations in clinical practice have been reported^31^. Watanabe et al. reported that extending the time to confirm functional patency to 72 h might be acceptable and increase the possibility to perform CE safely^25^. Meanwhile, Omori et al. reported that the 24-h assessment method can be handled more easily and more useful clinically^24^. Our modification in this study also proposes a highly feasible use of PC in daily practice.

This study has several limitations. Firstly, it is a single-centre, single-arm, retrospective study, although the participants were consecutive patients in clinical practice. Secondly, the overall patency rate was high in this study because we included many patients with OGIB in addition to CD. Therefore, the number of cases might be statistically insufficient for the analysis of factors related to non-confirmation of patency. Thirdly, since this study included inpatients and outpatients, it may be a selection bias if the SBCE is used in an outpatient routine. We confirmed that the bias did not significantly affect the interpretation of the overall results. The differences in target diseases between outpatients and inpatients were shown in Supplementary Table S2, and the results of the PC examination in outpatients only in Supplementary Table S3. Despite some limitations, our study certainly suggests that our modified criteria play a role in the real-world use of PCs in actual clinical practice. A prospective comparative study with a larger sample size and in multiple centres is required to verify these issues.

## Methods

### Study design

This single-centre, retrospective study conformed to the principles of the Declaration of Helsinki. In accordance with the Ethical Guidelines for Medical and Health Research Involving Human Subjects (Ministry of Education, Culture, Sports, Science and Technology and Ministry of Health, Labour and Welfare, Japan), study information including the objectives was disclosed on our hospital website with an opt-out approach. The Ethics Committee of Hamamatsu University School of Medicine in Japan reviewed and approved the study protocol (20-354).

We enrolled consecutive patients who underwent PC before SBCE between September 2012 and February 2020 in our hospital. The inclusion criteria were patients with suspected small-bowel stenosis scheduled for PC-based evaluation before SBCE. Physicians reviewed the small-bowel stenosis according to the patients’ medical records and interview. In particular, patients with stenotic symptoms but with unclear stenosis on imaging studies were enrolled. The exclusion criteria included patients with ongoing small-bowel obstruction, barium allergy and dysphagia. The indication for PC and SBCE examination, procedure, risks and countermeasures against potential complications were explained to each patient, and written informed consent was obtained.

### PC and SBCE procedures

We used the tag-less PillCam™ PC (Covidien Japan, Medtronic, Japan), which has the same size and components as the conventional Agile™ PC with a radiofrequency identification tag. After 12 h of fasting, patients swallowed the PC with water at 9 AM. After 2 h, drinking water was allowed, and in the next 2 h, a meal was provided. Excretion of the PC was confirmed visually using a PC recovery kit during bowel movement until their outpatient visit the following evening. Small-bowel patency was confirmed with a PC 30–33 h after its ingestion. ‘Confirmed patency’ was defined as the visual verification of an intact capsule (body and timer plugs are virtually intact) or intact body (body is intact and hard, but timer plugs have eroded) once excreted within 30 h, or the entry of an intact capsule into the large bowel confirmed by plain X-ray examination and further examination by plain CT, as necessary. Furthermore, ‘estimated patency’ was defined as the lack of PC evidence in the body via X-ray during the judgement time (30–33 h after ingestion). Within 7 days, patients with confirmed and estimated patency underwent SBCE (PillCam™ SB, Covidien Japan, Medtronic, Japan).

### Endpoints

The primary endpoint was the ‘confirmed patency’ and ‘estimated patency’ rates evaluated by small-bowel examination with PC, and then the SBCE retention rate. The secondary endpoints were the CT judgement rate, adverse events, factors associated with non-confirmation of patency and factors associated with excretion within 30 h.

### Statistical analysis

All statistical data were analysed using SPSS for Windows, version 16.0 (SPSS Inc., Chicago, Illinois, USA) and EZR (Saitama Medical Centre, Jichi Medical University, Saitama, Japan). Categorical data were evaluated using χ^2^-test and Fisher’s exact test. Factors associated with the nonconfirmation of PillCam™ PC-based gastrointestinal patency were identified by univariate and multivariate logistic regression analyses, and the results are expressed as crude and adjusted odds ratios (ORs) with 95% confidence intervals (CIs). The relationship between the period of time from PC ingestion to patency confirmation and the total enteroscopy rate was analysed using the Mann–Whitney *U* test. Differences were considered significant if their P values were less than 0.05. PC is useful for examining gastrointestinal patency prior to SBCE in clinical practice. In the judgement of patency, if the PC cannot be detected in the body by X-ray, performing SBCE as ‘estimated patency’ seems appropriate. Although plain CT is commonly used after PC detection via X-ray, physicians should be aware that the PC is difficult to locate accurately.

## Supplementary Information

Supplementary Tables.

## Abbreviations

SBCE: Small-bowel capsule endoscopy
OGIB: Obscure gastrointestinal bleeding
CD: Crohn’s disease
NSAID: Nonsteroidal anti-inflammatory drug
IBD: Inflammatory bowel disease
BAE: Balloon-assisted enteroscopy
PC: Patency capsule
CT: Computed tomography
