# Supplementary material for: Modified method of patency judgement using patency capsule prior to capsule endoscopy in clinical practice
Source: Sci Rep. 2022 Aug 22;12:14335. doi: 10.1038/s41598-022-18569-y (PMC9395361; doi:10.1038/s41598-022-18569-y)
Supplement: Supplementary file 1 — Supplementary Tables. [file 41598_2022_18569_MOESM1_ESM.docx]

**Modified method of patency judgement using patency capsule prior to capsule endoscopy in clinical practice**

Takahiro Miyazu^1^, Satoshi Osawa^2^, Satoshi Tamura^1^, Shinya Tani^1^, Natsuki Ishida^2^, Tomoharu Matsuura^1^, Mihoko Yamade^1^, Moriya Iwaizumi^3^, Yasushi Hamaya^1^, Takahisa Furuta^4^ and Ken Sugimoto^1^

^1^ First Department of Medicine, Hamamatsu University School of Medicine, Hamamatsu, Japan

^2^ Department of Endoscopic and Photodynamic Medicine, Hamamatsu University School of Medicine, Hamamatsu, Japan

^3^ Department of Laboratory Medicine, Hamamatsu University School of Medicine, Hamamatsu, Japan

^4^ Center for Clinical Research, Hamamatsu University School of Medicine, Hamamatsu, Japan

**Supplementary Information**

**Supplementary Table S1. List of patients judged to have no patency by patency test**

|  |  |  |  | Patency test | | | | Post-patency test | | |
| --- | --- | --- | --- | --- | --- | --- | --- | --- | --- | --- |
| Case | Age | M/F | Inpatient | Reason for examination | Location at CT | Cause of no patency | Adverse event | BAE | Final diagnosis | Treatment |
| 1 | 21 | M | No | Established CD | Ileum | Adhesion | Non | No | i-CD | Increase dose of immunomodulator |
| 2 | 49 | M | No | Established CD | Ileum | Stenosis | Non | Yes | i-CD | Continuation of medication |
| 3 | 25 | M | Yes | Established CD | Ileum | Stenosis | Non | Yes | i-CD | Follow-up |
| 4 | 34 | M | No | Established CD | Ileum | Stenosis | Non | No | ic-CD | Continuation of medication |
| 5 | 69 | M | No | Established CD | Ileum | Anastomic stenosis | Retention of the coating film | Yes | i-CD | Endoscopic balloon dilation |
| 6 | 63 | M | Yes | Established CD | Ileum | Intestinal obstruction | Non | Yes | c-CD | Continuation of medication |
| 7 | 30 | M | No | Established CD | Ileum | Stenosis | Non | Yes | ic-CD | Follow-up |
| 8 | 47 | M | No | Established CD | Ileum | Stenosis | Non | Yes | i-CD | Continuation of medication |
| 9 | 21 | M | Yes | Suspected CD | Ileum | Unknown | Non | No | c-CD | Change of anti-TNFα therapy |
| 10 | 69 | M | Yes | Suspected CD | Stomach | Gastroparesis | Non | Yes | AGML | Follow-up |
| 11 | 56 | F | No | NF-I, after surgery of GIST | Ileum | Stenosis | Vomiting | No | NF-I | Follow-up |
| 12 | 60 | M | Yes | OGIB, occult | Ileum | Stenosis | Non | Yes | Ileal ring ulcer | Mucosal protective drug (rebamipide) |
| 13 | 78 | M | Yes | OGIB, occult | Oesophagus | Oesophageal motility disorder | Non | No | No definitive diagnosis | Change of gastric acid secretion inhibitor |
| 14 | 81 | F | Yes | OGIB, overt | Ileum | Stenosis | Non | Yes | No definitive diagnosis | Small-bowel resection |
| 15 | 51 | F | Yes | Radiation enteritis | Ileum | Unknown | Non | Yes | Radiation enteritis | Small-bowel resection |
| 16 | 68 | F | Yes | Hematochezia | Ileum | Anastomotic stenosis | Non | No | Rectal angioectasia | Argon plasma coagulation for angioectasia |

AGML, acute gastric mucosal lesions; BAE, balloon-assisted enteroscopy; c-CD, colonic Crohn’s disease; CD, Crohn’s disease; i-CD, ileal Crohn’s disease; ic-CD, ileocolic Crohn’s disease; NF-I, neurofibromatosis type I; OGIB, obscure gastrointestinal bleeding

**Supplementary Table S2. Patients’ characteristics (outpatient vs inpatient)**

|  | outpatient | inpatient | p-value |
| --- | --- | --- | --- |
| Numbers of patients | 200 | 126 |  |
| Sex, male/female | 119/81 | 79/47 | n.s.^*^ |
| Age, mean ± SD (range), years | 53.2 ± 20.0 (14–88) | 50.7 ± 25.2 (3–85) | n.s^**^ |
| History of abdominal surgery, n (%) | 58 (29.0) | 50 (39.7) | n.s.^*^ |
| Reason for examination, n (%)  OGIB  Crohn’s disease, established  Crohn’s disease, suspected  Other inflammatory diseases  Abdominal pain  Small-bowel tumour  Intestinal obstruction  Others | 62 (31.0)  56 (28.0)  10 (5.0)  13 (6.5)  15 (7.5)  14 (7.0)  2 (1.0)  28 (14.0) | 55 (43.7)  19 (15.1)  6 (4.8)  16 (12.7)  12 (9.5)  6 (4.8)  3 (2.4)  9 (7.1) | <0.05^*^  <0.01^*^  n.s.^*^  n.s.^*^  n.s.^*^  n.s.^*^  n.s.^*^  n.s.^*^ |
| Diabetes mellitus | 22 (11.0) | 29 (23.0) | <0.01^*^ |
| Haemodialysis | 2 (1.0) | 17 (13.5) | <0.01^*^ |
| Constipation | 29 (14.5) | 18 (14.3) | n.s.^*^ |
| NSAIDs, LDA | 31 (15.5) | 33 (26.2) | <0.05^*^ |

LDA, low-dose aspirin; NSAIDs, nonsteroidal anti-inflammatory drugs; OGIB, obscure gastrointestinal bleeding; SD, standard deviation; n.s., not significant

^*^Fisher’s exact test　^**^Student t-test

**Supplementary Table S3. Results of the patency capsule procedure: outpatient**

| Overall patency, n (%) | 192 (96.0) |
| --- | --- |
| Confirmed patency, n (%) | 165 (82.5) |
| Estimated patency, n (%) | 27 (13.5) |
| CT judgement, n (%) | 49 (24.5) |
| No patency, n (%) | 8 (4.0) |
| Adverse events, n (%)  Retention of the coating film  Abdominal pain  Nausea, vomiting  Intestinal obstruction  Perforation  Capsule aspiration  Allergic reaction | 2 (1.0)  1 (0.5)  1 (0.5)  0 (0.0)  0 (0.0)  0 (0.0)  0 (0.0)  0 (0.0) |
